# Molecular Characterization of Oral Epithelial Dysplasia and Oral Squamous Cell Carcinoma Using EGFR, CDKN2A, and HRAS Alterations

**DOI:** 10.3390/cancers17243949

**Published:** 2025-12-10

**Authors:** Satoshi Okubo, Satoru Miyabe, Masahiro Fukumura, Jun Sasaki, Hitoshi Fujii, Fumitaka Terasawa, Satoshi Watanabe, Soma Okada, Megumi Miyabe, Katsuyuki Miyabe, Yoshihiko Sugita, Hatsuhiko Maeda, Sanako Nakaya, Kaori Sakane, Seiji Yamada, Nitin Bhola, Saman Warnakulasuriya, Toru Nagao, Mitsuo Goto

**Affiliations:** 1 Department of Oral and Maxillofacial Surgery, School of Dentistry, Aichi Gakuin University, Nagoya 464-8651, Japan; ag223d06@dpc.agu.ac.jp (S.O.); masahiro_fukumura@mail.toyota.co.jp (M.F.); jun0626@dpc.agu.ac.jp (J.S.); h-fujii@dpc.agu.ac.jp (H.F.); terafumi@dpc.agu.ac.jp (F.T.); s-nabeo@dpc.agu.ac.jp (S.W.); okaoka34@dpc.agu.ac.jp (S.O.); ag233d13@az.agu.ac.jp (S.N.); kaori.u.0201@gmail.com (K.S.); tnagao@dpc.agu.ac.jp (T.N.); mgoto@dpc.agu.ac.jp (M.G.); 2 Department of Internal Medicine, School of Dentistry, Aichi Gakuin University, Nagoya 464-8651, Japan; mmiyabe@dpc.agu.ac.jp; 3 Department of Gastroenterology, Japanese Red Cross Aichi Medical Center Nagoya Daini Hospital, Nagoya 466-8650, Japan; kmiyabe@nagoya2.jrc.or.jp; 4 Department of Oral Pathology/Forensic Odontology, School of Dentistry, Aichi Gakuin University, Nagoya 464-8650, Japan; yosshii@dpc.agu.ac.jp (Y.S.); hatsu@dpc.agu.ac.jp (H.M.); 5 Division of Analytical Pathology, Oncology Innovation Center, Research Promotion Headquarters, Fujita Health University School of Medicine, Toyoake 470-1192, Japan; yamadas@fujita-hu.ac.jp; 6 Department of Oral & Maxillofacial Surgery, Sharad Pawar Dental College, Datta Meghe Institute of Medical Sciences, Wardha 442001, India; drnitinbhola@gmail.com; 7 Faculty of Dentistry, Oral and Craniofacial Sciences, King’s College London, WHO Collaborating Centre for Oral Cancer, London SE1 9RT, UK; saman.warne@kcl.ac.uk

**Keywords:** CDKN2A, EGFR, gene mutation, head and neck neoplasms, HRAS, oral epithelial dysplasia, oral squamous cell carcinoma, prognosis

## Abstract

**Background/Objectives:** Oral squamous cell carcinoma (OSCC) often presents at an advanced stage; therefore, the early detection of precursor lesions is crucial. However, the risk assessment of precursor lesions such as oral epithelial dysplasia (OED) remains challenging because of the subjectivity of histopathological grading. We aimed to identify molecular markers that enhance the diagnostic accuracy and prognostic stratification of OSCC and explore the differences in the molecular characterization of OED and OSCC using a few selected markers. **Methods:** A two-step diagnostic workflow was applied: (1) FISH evaluation of *EGFR* amplification and *CDKN2A* deletion to distinguish OED from OSCC and identify *EGFR*-dependent tumors, and (2) HRAS immunohistochemistry performed exclusively in *EGFR*-negative OSCCs to stratify *EGFR*-independent cases. Fluorescence in situ hybridization (FISH) was used to assess seven *EGFR*/cell cycle-related genes (*CCND1*, *CDKN2A*, *EGFR*, *PIK3CA*, *PTEN*, *TP53*, and 1p36 locus) in 117 formalin-fixed paraffin-embedded samples (66 OED and 51 OSCC) and 10 normal mucosa samples. HRAS expression was evaluated using immunohistochemistry (IHC) in 36 *EGFR* amplification-negative OSCCs samples. **Results:** *EGFR* amplification was frequent in OSCC, whereas *CDKN2A* deletion was common in OED. The *EGFR*-amplified/ *CDKN2A*-intact profile showed high specificity for OSCC and improved diagnostic performance (area under the curve = 0.77) when combined with the Ki-67 labeling index. It also predicted poor disease-free survival (hazard ratio [HR] = 5.08, *p* = 0.016) and overall survival (HR = 6.10, *p* = 0.047). Among *EGFR*-negative OSCCs, HRAS overexpression was associated with advanced-stage disease and a poor prognosis (HR = 6.15, *p* = 0.043). **Conclusions:** *EGFR* amplification was frequent in OSCC, and *CDKN2A* deletion was prevalent in OED, supporting their use as molecular markers for differential diagnoses. FISH for *EGFR/CDKN2A* and HRAS IHC can stratify OSCC by diagnosis and prognosis, enabling practical molecular subclassification, including *EGFR*-negative cases.

## Simple Summary

We tested molecular markers to improve early diagnosis and risk assessment of patients diagnosed with oral cancer. Using routine biopsy tissue, the application of FISH technique for EGFR amplification and CDKN2A deletion distinguished oral squamous cell carcinoma from non-cancerous dysplasia with high specificity, improved diagnostic performance, and identified patients with poorer survival. In cancers without EGFR amplification, high HRAS expression indicated aggressive disease and unfavorable prognosis. This two-step biomarker panel (EGFR/CDKN2A by FISH, then HRAS IHC if EGFR-negative) may help clinicians decide which oral lesions require aggressive treatment and closer follow-up.

## 1. Introduction

Oral squamous cell carcinoma (OSCC) is the most common subtype of head and neck squamous cell carcinoma (HNSCC) and is among the 16 most prevalent cancers worldwide [1,2]. In 2022, an estimated 380,000 new cases of OSCC and approximately 180,000 OSCC-related deaths were reported globally [3]. Despite advances in surgical and systemic therapies, the prognosis of patients with advanced-stage OSCC remains poor, with a 5-year survival rate of approximately 50% [4,5]. This is largely attributed to the delayed diagnosis and high locoregional recurrence rates. Therefore, early detection of malignant transformation of oral potentially malignant disorders, such as leukoplakia and erythroplakia, is crucial for improving survival outcomes [6,7].

Oral leukoplakia and erythroplakia, with or without oral epithelial dysplasia (OED), are premalignant lesions that can progress to OSCC. However, accurate diagnosis at an early malignant stage is particularly challenging and currently relies on the subjective histopathological assessment of biopsy samples using hematoxylin–eosin (HE)-stained sections. Owing to interobserver variability and biological heterogeneity of lesions, the predictive accuracy of such assessments is limited. Consequently, the reported malignant transformation rate of OED ranges from 4% to 30% [8,9]. Currently, there are few reliable molecular markers tested for use that can be detected using immunohistochemistry (IHC) for risk prediction; however, they lack good sensitivity [10]. This gap highlights the urgent need for reliable molecular biomarkers to support early and precise diagnosis [11].

Advances in molecular oncology have revealed key genetic alterations in OSCC, including mutations in the tumor protein *p53 (TP53)*, phosphatidylinositol-4,5-bisphosphate 3-kinase catalytic subunit alpha *(PIK3CA)*, cyclin dependent kinase inhibitor 2A *(CDKN2A)*, and epidermal growth factor receptor *(EGFR)* [12]. *EGFR* regulates cell proliferation, survival, and motility via the RAS/MAPK and PI3K/AKT pathways [13]. Its overexpression and amplification are linked to tumor progression, and anti-*EGFR* therapies such as cetuximab are being tested for advanced HNSCC. However, their efficacy in OSCC remains variable [3,14]. Other alterations, including *CDKN2A* deletion and *Cyclin D1 (CCND1)* amplification, also have therapeutic relevance to the CDK4/6 inhibitors under investigation [15]. Although EGFR-targeted therapy is an important component of OSCC treatment in many regions [16], *EGFR* acts as a driver in only 20–40% of cases [3,13]. Alternative oncogenic drivers in *EGFR*-negative tumors remain unclear, highlighting the need for comprehensive molecular profiling and identification of new therapeutic targets.

Genes related to *EGFR* and cell cycle, such as *CDKN2A* and *TP53*, have shown promise for diagnostic and prognostic stratification [2,17]. Additionally, deletion of the 1p36 chromosomal locus, which harbors multiple tumor suppressor genes such as *TP73*, has been associated with early genomic instability and may aid in OSCC detection [18]. Therefore, 1p36 deletion was included in this study to assess its role in molecular stratification.

Recent evidence regarding non-small cell lung cancer suggests that co-alterations in *EGFR* and *CDKN2A* may enhance oncogenicity and reshape the immune microenvironment. This combination promotes PD-L2 expression and immune evasion and sensitizes tumors to *EGFR*-tyrosine kinase inhibitors and immune checkpoint blockade [19]. Based on lung cancer data, we hypothesized that this molecular profile is relevant in OSCC.

Harvey Rat Sarcoma Viral Oncogene Homolog (HRAS), a key MAPK pathway activator, is mutated in 1.2–18% of OSCCs depending on population and method [20,21] and may contribute to *EGFR*-independent progression. However, the clinical significance of HRAS expression, particularly in *EGFR*-negative OSCC, remains unclear and warrants further study [22].

To explore the differences in molecular alterations between premalignant and malignant lesions, we first performed comprehensive fluorescence in situ hybridization (FISH) analysis of seven *EGFR* pathway-related driver genes (*CCND1, CDKN2A, EGFR, PIK3CA, PTEN, TP53*, and 1p36 locus) in both the OED and OSCC cohorts. Among these, *EGFR* amplification and *CDKN2A* deletion emerged as key alterations that significantly distinguished OSCC from OED. Based on this finding, we focused on *EGFR* amplification-negative OSCCs, which lack established molecular classification frameworks and have limited treatment options. To explore alternative oncogenic pathways and potential prognostic markers in this subgroup, we assessed HRAS expression using IHC, based on its role in MAPK signaling and its therapeutic relevance.

Therefore, we used a two-tiered strategy: (1) FISH-based profiling to distinguish OED from OSCC and (2) IHC-based stratification of *EGFR*-negative OSCCs using HRAS. Using this approach, we aimed to enhance the diagnostic precision, establish a practical framework for the molecular subclassification of OED and OSCC, and provide treatment guidance for OSCC.

## 2. Materials and Methods

### 2.1. Patient Samples and Ethical Approval

This retrospective study was approved by the Ethics Committee of the Aichi Gakuin University (approval no. 98). Formalin-fixed paraffin-embedded (FFPE) tissue specimens were obtained from 66 patients with oral leukoplakia with epithelial dysplasia and 51 patients with OSCC treated at the Aichi Gakuin University. Diagnoses were confirmed by three board-certified oral pathologists, according to the 5th edition of the World Health Organization classification (2022) [23]. Eligible patients included those with histopathological diagnoses of OED or OSCC and available clinical data (sex, age, and lesion site). The exclusion criteria were insufficient tissue for histology or FISH, prior radiotherapy or chemotherapy, distant metastasis, or incomplete TNM staging. This study was conducted at the Aichi Gakuin University Dental Hospital between October 2003 and December 2022. Of 123 initially identified FFPE samples, six were excluded: three owing to inadequate tissue, two owing to prior treatment, and one owing to missing clinical data. A total of 117 patients (66 with OED and 51 with OSCC) were included in the final analysis. Additionally, 10 normal mucosal samples were collected from areas adjacent to the tumor to avoid direct tumor contact and minimize contamination. Normality was confirmed using HE staining, and the samples were analyzed using the same FISH protocol as that used for the tumor specimens.

### 2.2. Fluorescence In Situ Hybridization

FISH was performed on 4 μm-thick FFPE tissue sections using commercially available dual-color probes (ZytoVision, Bremerhaven, Germany) targeting *CCND1*/CEP11, *CDKN2A*/CEP9, *EGFR*/CEP7, *PIK3CA*/CEP3, *PTEN*/CEP10, *TP53*/CEP17, and 1p36/1q25. The 1p36 probes included *TP73* and *EGFL3*, and the 1q25 controls included *ABL2* and *ANGPTL1*. We followed standardized protocols [24]. Briefly, slides were deparaffinized, rehydrated, and enzymatically digested with 4% pepsin at 37 °C for 15 min. After denaturation at 70 °C for 2 min in 70% formamide/2 × SSC, hybridization was performed overnight at 37 °C in a humidified chamber. Following stringent post-hybridization washes, the nuclei were counterstained with 4′,6-diamidino-2-phenylindole and mounted with antifade medium. Fluorescence signals were evaluated using an Olympus BX51 fluorescence microscope and analyzed using the Applied Spectral Imaging software (cellSens® V1.11). To minimize bias and improve reproducibility, two observers (S.M. and S.Y.), blinded to the clinical and histological data, independently interpreted the signals. At least 200 interphase nuclei were assessed per case, and discrepancies were resolved by consensus. Copy number alterations were defined as follows: amplification, a gene/centromere ratio ≥ 2.0 or >10 signals per nucleus in ≥20% of tumor cells; deletion, loss of one or both gene signals in ≥30% of tumor cells [24]. Due to nuclear truncation and signal overlap inherent to FFPE tissue sections, the FISH assay used in this study primarily detects hemizygous loss. Distinguishing hemizygous from biallelic deletion was not technically feasible; therefore, deletion was defined as the loss of one or both gene signals, consistent with standard FISH criteria. Lymphocytes served as internal diploid controls. In addition to gene-level assessments, cases with combined *EGFR* amplification and *CDKN2A* deletion (*EGFR amp/CDKN2A intact*) were identified and further analyzed for prognostic relevance.

### 2.3. HRAS IHC in EGFR-Negative OSCC

Following the initial FISH-based profiling of 117 cases, all OSCC specimens were subjected to Ki-67 IHC (MIB-1, 1:100, Dako, Copenhagen, Denmark) to assess the proliferative activity and assist in histological grading. Among the 51 patients with OSCC, 36 lacked *EGFR* amplification. Notably, all *EGFR*-negative tumors lacked *CDKN2A* deletions, suggesting a molecularly distinct subgroup that is not covered by the current classifications or EGFR-targeted therapies. To explore alternative oncogenic drivers and prognostic markers in this group, HRAS IHC was performed using a polyclonal antibody (1:200; Proteintech, Rosemont, IL, USA; #18295-1-AP). Staining was performed on the Bond-Max automated system (Leica Microsystems, Wetzlar, Germany) using the Bond Polymer Refine Detection Kit. HRAS immunoreactivity was semiquantitatively scored using a modified Allred system, with reference to the HE-stained slides. Scores combined the staining intensity (0–3) and positive cell proportion (0–5), yielding a total score of 0–8 [22]. Expression levels were classified as 0 (negative), 2–3 (weak), 4–6 (moderate), and 7–8 (strong). For statistical analysis, scores of 0–3 and 4–8 were defined as low and high, respectively [22]. All IHC slides were independently reviewed by two board-certified pathologists (S.M. and Y.S.), and discrepancies were resolved by consensus.

### 2.4. Statistical Analysis

The clinical and pathological data were summarized descriptively. Associations between genetic alterations and clinicopathological factors were evaluated using the chi-square or Fisher’s exact tests. Continuous variables were compared using the Mann–Whitney U or Kruskal–Wallis tests. Diagnostic performance was assessed using the receiver operating characteristic (ROC) curve analysis (sensitivity, specificity, and area under the curve [AUC]). Overall survival (OS) and disease-free survival (DFS) were estimated using the Kaplan–Meier method, and differences between groups were compared using log-rank tests. Multivariate survival analysis was performed using the Cox proportional hazards model. Two-sided *p*-values < 0.05 were considered significant. All analyses were performed using the JMP software (v14.2, SAS Institute, Cary, NC, USA).

## 3. Results

**Figure 1:**
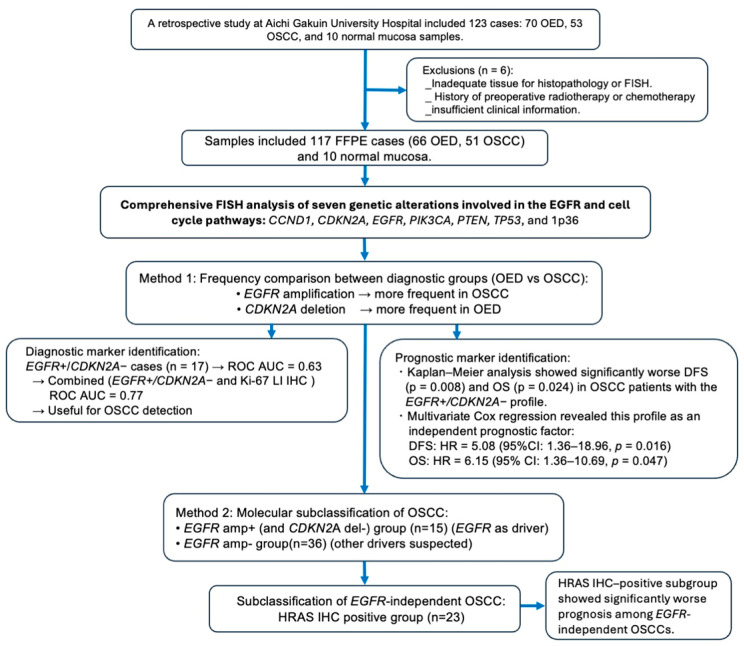
Flowchart of the study design and molecular classification of OSCC. This flowchart outlines the study process using 117 FFPE samples (66 OED, 51 OSCC, and 10 normal mucosa samples). FISH analysis targeting seven genes revealed that *EGFR* amplification was more frequent in OSCC than in OED, whereas *CDKN2A* deletion was more frequent in OED than in OSCC. The *EGFR amp /CDKN2A intact* profile was associated with significantly poor DFS and OS and was identified as an independent prognostic factor using the Cox proportional hazards model. Based on FISH and IHC findings, OSCC was subclassified into *EGFR*-dependent and *EGFR*-independent groups, with an HRAS IHC-positive subgroup showing a poor prognosis among *EGFR*-independent OSCCs. Abbreviations: amp+, amplification-positive; del−, deletion-negative; LI, labeling index; OSCC, oral squamous cell carcinoma; OED, oral epithelial dysplasia; ROC, receiver operating characteristic; AUC, area under the ROC curve; HR, hazard ratio; CI, confidence interval; IHC, immunohistochemistry; FISH, fluorescence in situ hybridization; FFPE, formalin-fixed paraffin-embedded.

We assessed the clinicopathological profiles and genetic alterations in 127 samples: 10 normal oral mucosa, 66 OED, and 51 OSCC (Table 1). The overall study design and analytical workflow are shown in Figure 1.

**Figure 2:**
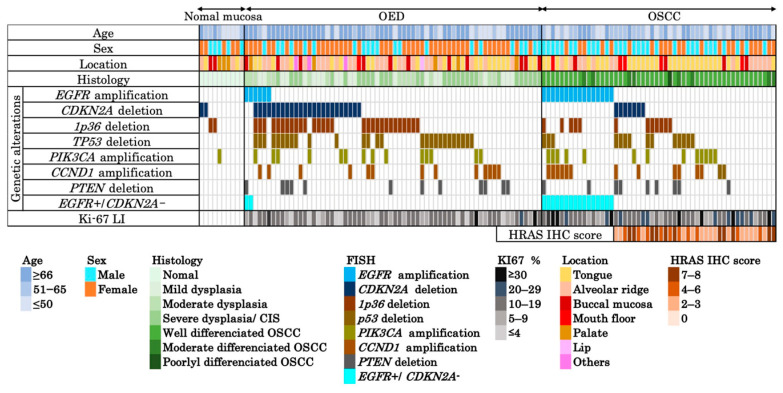
Integrated clinicopathological and molecular profiles across normal mucosa, OED, and OSCC. Heatmap summarizing the clinical (age, sex, and location), histological, and molecular features of 117 formalin-fixed paraffin-embedded samples (10 normal mucosa, 66 OED, 51 OSCC). Genetic alterations were assessed using FISH targeting *EGFR, CDKN2A*, 1p36 locus, *TP53, PIK3CA, CCND1*, and *PTEN*, listed from top to bottom in descending order of frequency. A combined profile of *EGFR* amplification and *CDKN2A* deletion negativity (*EGFR amp/CDKN2A intact*), specific to OSCC, is separately indicated. The Ki-67 LI is shown for all cases. HRAS IHC scores (range: 0–8) were evaluated in 36 OSCC cases lacking *EGFR* amplification. Each column represents a single case. Abbreviations: OSCC, oral squamous cell carcinoma; OED, oral epithelial dysplasia; CIS, carcinoma in situ; LI, labeling index; IHC, immunohistochemistry.

FISH analysis targeted seven genes related to EGFR signaling and cell cycle regulation: *CCND1, CDKN2A, EGFR, PIK3CA, PTEN, TP53*, and 1p36 locus. The results are summarized in Supplementary Table S1 and shown in Figure 2. *EGFR* amplification was more frequent in OSCC than in OED (29.4% vs. 9.1%; *p* = 0.007), whereas *CDKN2A* deletion was more frequent in OED than in OSCC (36.4% vs. 13.7%; *p* = 0.006) (Table 2). No significant group differences were found in *CCND1* amplification (25.5% vs. 19.7%; *p* = 0.51), *TP53* deletion (27.5% vs. 40.9%; *p* = 0.17), *PTEN* deletion, or *PIK3CA* amplification. In contrast, 1p36 deletion was significantly more common in OED (43.9%) than in OSCC (21.6%; *p* = 0.018), suggesting its role in dysplasia. None of the seven alterations showed a significant prognostic association with OSCC. We also examined whether CDKN2A deletion status or the presence of 1p36 deletion was associated with the clinicopathologic variables in Table 1. In both OED and OSCC, neither alteration showed significant correlations with age, sex, anatomical or tumor site, dysplasia or histological grade, or clinical stage (all *p* > 0.05).

### 3.1. FISH Analysis of EGFR amp/CDKN2A Intact Profile for Diagnosis and Prognosis of Patients with OSCC

**Figure 3:**
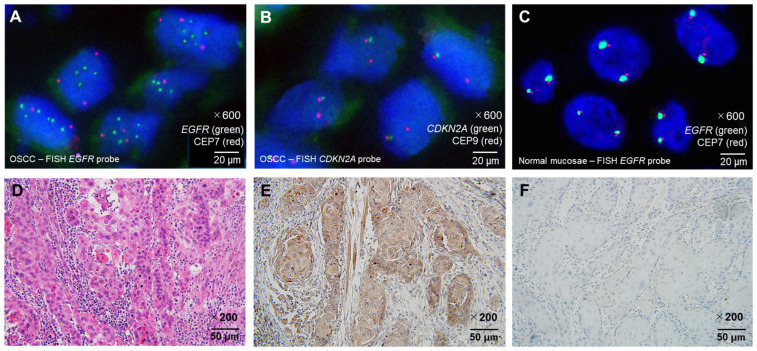
Representative histopathological and molecular findings of OSCC and normal mucosa. (**A**) FISH image showing EGFR gene amplification in OSCC, characterized by increased green EGFR locus signals relative to red CEP7 centromeric signals (×600 magnification). (**B**) Representative example of CDKN2A deletion in OSCC by FISH, demonstrating loss of one green CDKN2A locus signal with preservation of the red CEP9 centromeric signals (×600 magnification). Note that CDKN2A deletion is overall more frequent in OED than in OSCC. (**C**) FISH image of normal tongue mucosa showing no EGFR amplification, with green EGFR and red CEP7 signals present in normal copy numbers (×600 magnification). (**D**) Hematoxylin and eosin staining of well-differentiated OSCC (×200 magnification). (**E**) HRAS protein overexpression detected by immunohistochemistry (IHC) in the same OSCC case shown in (**D**) (×200 magnification). (**F**) Low HRAS expression detected by IHC in OSCC (×200 magnification). Probe colors: EGFR = green; CDKN2A = green; CEP7 = red; CEP9 = red. Scale bars are indicated in each panel. Abbreviations: OSCC, oral squamous cell carcinoma; OED, oral epithelial dysplasia; IHC, immunohistochemistry; FISH, fluorescence in situ hybridization.

**Figure 4:**
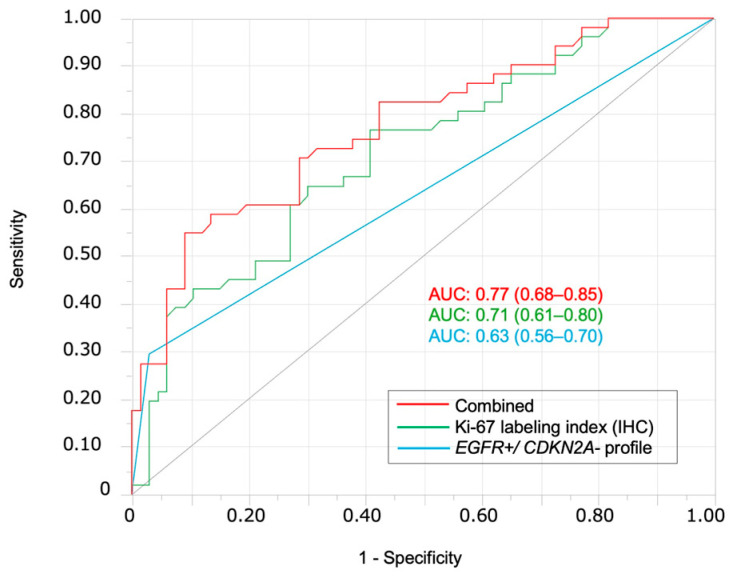
ROC curves for differentiating OSCC from OED. ROC curves demonstrate the diagnostic performance of the *EGFR amp/CDKN2A intact* profile alone (blue, AUC = 0.63), Ki-67 labeling index alone (green, AUC = 0.71), and their combination (red, AUC = 0.77). The combination shows the highest diagnostic accuracy for distinguishing OSCC from OED; AUC, area under the ROC curve.

**Figure 5:**
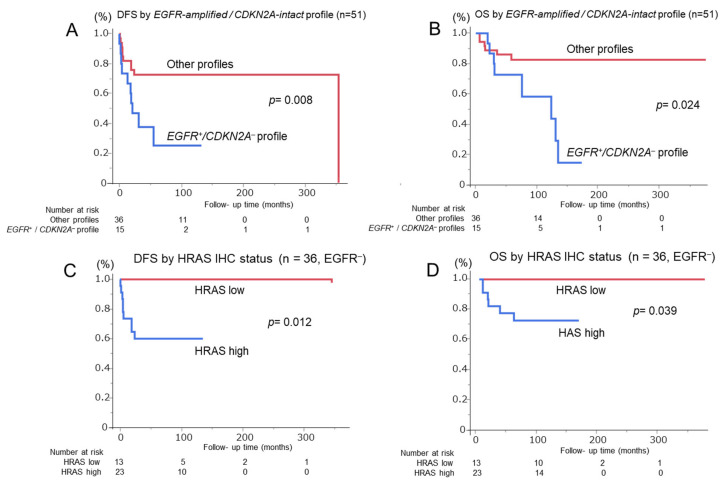
Kaplan–Meier survival curves for patients with OSCC stratified by molecular profiles. (**A**,**B**) Disease-free survival (DFS) and overall survival (OS) of 51 OSCC cases according to the *EGFR* amplification-positive/*CDKN2A* deletion-negative (*EGFR amp/CDKN2A intact*) profile. Patients with this profile show significantly worse DFS (*p* = 0.008) and OS (*p* = 0.024) than those without. (**C**,**D**) DFS and OS of 36 *EGFR*-negative OSCC cases stratified by HRAS IHC status. High HRAS expression is significantly associated with poor DFS (*p* = 0.012) and OS (*p* = 0.039) compared with low HRAS expression. Number at risk is indicated below each graph. Abbreviations: DFS, disease-free survival; OS, overall survival; IHC, immunohistochemistry.

The *EGFR* amplification-positive and *CDKN2A* deletion-negative *(EGFR amp/CDKN2A intact*) profile was evaluated as a diagnostic marker to distinguish OSCC from OED. This profile was found in 15 OSCC cases (29.4%) but only in 2 OED cases (3.0%) (*p* < 0.0001) (Figure 3). *EGFR* amplification was observed in 6 of 66 OED cases (9.1%), including 2 patients with mild dysplasia and 3 patients with moderate dysplasia; the remaining patient later developed metachronous squamous cell carcinoma and died of tumor progression. Logistic regression analysis confirmed that it was an independent predictor of OSCC (*p* = 0.0003; Table 2). ROC analysis yielded an AUC of 0.63 (sensitivity, 29.4%; specificity, 97.0%; and accuracy, 67.5%). When combined with the Ki-67 labeling index, the AUC improved to 0.77 (sensitivity, 54.9%; specificity, 90.9%; and accuracy, 75.2%) (Figure 4). Adding the 1p36 deletion further increased the AUC to 0.80 and sensitivity to 76.5%, although specificity declined to 75.8%, and overall accuracy improved only slightly to 76.1%. The Kaplan–Meier analysis showed significantly poor DFS (*p* = 0.008) and OS (*p* = 0.024) in *EGFR amp/CDKN2A intact* OSCCs (Figure 5A,B). The multivariate Cox analysis confirmed its prognostic significance (DFS: hazard ratio [HR] = 5.08, *p* = 0.016; OS: HR = 6.10, *p* = 0.047; Table 3). No *EGFR amp/CDKN2A intact* cases were observed in the 10 normal mucosa samples, although low-frequency alterations in *PIK3CA* (10%), *CDKN2A* (20%), and 1p36 (20%) were detected (Table 1 and Figure 2 and Figure 3C).

### 3.2. Prognostic Impact of HRAS Expression in EGFR-Negative OSCC

After confirming the diagnostic relevance of the *EGFR amp/CDKN2A intact* profile, OSCC cases were stratified by *EGFR* amplification status. All *EGFR*-amplified cases lacked the *CDKN2A* deletion, supporting the exclusivity of this molecular pattern (Figure 2). To further classify *EGFR*-negative OSCCs, HRAS expression was analyzed owing to its downstream role in EGFR signaling. HRAS immunoreactivity was semiquantitatively scored using the modified Allred system. Of the 51 OSCCs, 36 (70.6%) were found to be *EGFR*-negative. Among them, HRAS expression was high in 23 cases and low in 13 (Figure 3D–F). High HRAS expression (scores 4–8) was correlated with advanced tumor features, including large pathological size (*p* = 0.01), high TNM stage (*p* = 0.004), and the need for neck dissection (*p* = 0.030) (Supplementary Table S2). The Kaplan–Meier analysis showed significantly shorter DFS (*p* = 0.012) and OS (*p* = 0.039) in high-HRAS cases than in low-HRAS cases (Figure 5C,D). Similarly, clinical stages III–IV were associated with poor survival (DFS: *p* = 0.002; OS: *p* = 0.038; Table 4). The multivariate Cox analysis confirmed that high HRAS expression was an independent predictor of poor prognosis for both DFS (HR = 5.91, *p* = 0.022) and OS (HR = 6.15, *p* = 0.043).

## 4. Discussion

Histopathologically, distinguishing severe oral epithelial dysplasia (OED) from superficially invasive or microinvasive oral squamous cell carcinoma (OSCC) remains challenging because minimal stromal invasion can be difficult to identify [25]. This diagnostic overlap contributes to interobserver variability and highlights the need for objective molecular markers to support more reliable differentiation. Although several biomarkers, such as Ki-67, p53, SOX2, podoplanin, and DNA aneuploidy, have been proposed, only Ki-67 and p53 are routinely used and have limited diagnostic reliability [26].

In this study, *EGFR* amplification was frequent in OSCC, whereas *CDKN2A* deletion was common in OED, supporting their roles as molecular markers for differential diagnoses. None of the OSCC cases showed co-occurrence, whereas some OED cases exhibited co-occurrence, suggesting distinct oncogenic pathways (Figure 2). The *EGFR amp/CDKN2A intact* profile was specific to OSCC and showed high diagnostic accuracy in ROC analysis. The diagnostic performance was further improved when combined with the Ki-67 labeling index, highlighting the complementary utility of FISH and IHC [27]. Although Ki-67 alone demonstrated higher diagnostic sensitivity than the *EGFR amp/CDKN2A intact* profile, the two markers capture biologically distinct aspects of lesion behavior. Ki-67 reflects proliferative activity, which may increase in reactive atypia or inflamed lesions, whereas EGFR amplification and CDKN2A deletion represent early molecular driver events specific to OSCC development. Therefore, their combination provided higher diagnostic specificity and improved overall diagnostic performance. The limited number of *EGFR amp/CDKN2A intact* OED cases (n = 2) restricts the statistical power of subgroup analyses, and this limitation has been acknowledged. Adding the 1p36 deletion increased the AUC and sensitivity but reduced the specificity, reflecting a trade-off between early instability detection and diagnostic precision. FISH for *EGFR* and *CDKN2A* was applicable to the FFPE samples and provided reproducible results, particularly for *EGFR* [28]. *CDKN2A* deletion may also help screen for high-risk OED and improve the diagnostic accuracy [29]. *EGFR* amplification was also observed in six OED cases (two mild, three moderate, and one severe). Among these cases, one patient with severe OED showing EGFR amplification later developed OSCC at both the original dysplastic site and another distinct oral site. While only the former represents true malignant transformation, the latter metachronous tumor likely reflects field cancerization or a high-risk mucosal field. This case highlights that EGFR-amplified severe OED warrants careful long-term surveillance. In our cohort, EGFR amplification and CDKN2A deletion were mutually exclusive in OSCC, which limits the ability to evaluate the independent prognostic contribution of CDKN2A loss within EGFR-amplified tumors. Consequently, the prognostic effect observed for the *EGFR-amplified/CDKN2A-intact* profile largely reflects the impact of EGFR amplification alone. This mutual exclusivity pattern may be unique to our patient population, and the prognostic utility of this combined profile requires validation in larger external cohorts, including public datasets that contain tumors with coexisting EGFR amplification and CDKN2A deletion.

FISH analysis of 10 normal mucosal samples revealed no alterations in *EGFR, TP53, CCND1*, or *PTEN*, whereas sporadic aberrations were observed in *PIK3CA* (10%), *CDKN2A* (20%), and 1p36 (20%), consistent with previously reported age-related genomic changes [30,31]. Importantly, the *EGFR amp/CDKN2A intact* profile was absent in all the normal samples, reinforcing its specificity for OSCC. These findings underscore the diagnostic value of assessing combined molecular alterations rather than individual markers, as proposed in a previous study by Monteiro et al. [32]. Notably, the 1p36 deletion was more frequent in OED than in OSCC, supporting its potential as an early marker of genomic instability [18]. Building on the diagnostic stratification achieved using *EGFR* and *CDKN2A* FISH, we focused on *EGFR*-negative OSCCs, which lack the known molecular drivers. To explore the potential prognostic indicators and therapeutic targets in this subgroup, we examined HRAS expression using IHC.

*CDKN2A* deletion was more prevalent in OED than in OSCC (36.4% vs. 13.7%), contrary to previous reports [29]. Given the role of *CDKN2A* in early cell cycle regulation and frequent alterations in head and neck cancers [33], its presence in OED is biologically plausible. Shahnavaz et al. reported homozygous deletions in 12% of OED and 78% of OSCC cases using laser microdissection, suggesting stepwise progression [29]. Similarly, Kojima et al. identified *CDKN2A* mutations in premalignant lesions [34]. These findings support the clonal evolution model observed in head and neck cancers [35] and other precancerous conditions, such as colorectal adenomas and cervical intraepithelial neoplasia [36]. Although deletion of CDKN2A and 1p36 was significantly more frequent in OED, our FISH assay primarily detects hemizygous loss rather than true biallelic deletion. Therefore, the biological implications of single-copy versus two-copy loss could not be evaluated in this study. In our cohort, the high prevalence of *CDKN2A* deletions in OED may reflect early hemizygous loss, which is preferentially detected using FISH. In contrast, homozygous deletions, which are indicative of irreversible clonal dominance, may be underdetected and require complementary methods for confirmation [30,31]. This observation supports a nonlinear evolutionary model in which some *CDKN2A*-deleted clones may undergo clonal extinction [37]. Given the molecular heterogeneity of OED, *CDKN2A* deletion may indicate a high-risk but transient intermediate state [38]. In OSCC, *CDKN2A* deletion was found in 7 of 36 *EGFR*-negative cases, most of which were well-differentiated (5/7) and early stage (4/7) and had a 5-year survival rate of 71.4%, comparable to *CDKN2A*-intact cases (n = 29). This suggests a limited prognostic value in this context, possibly because of the predominance of low-grade tumors. Overall, these findings highlight the complexity of *CDKN2A* in oral carcinogenesis and underscore the need for an integrated evaluation using both deletion-sensitive and functionally informative approaches. In addition, CDKN2A and 1p36 deletions showed no association with any clinicopathologic variables in either OED or OSCC, suggesting that these alterations may arise independently of conventional phenotypic features. This supports the view that such deletions represent early or parallel molecular events rather than progression markers linked to clinical stage or tumor differentiation.

Cetuximab, an anti-EGFR monoclonal antibody, is approved for advanced OSCC and HNSCC and offers a therapeutic option for EGFR-positive tumors, although its response rate remains limited to 10–20% [39]. Tumors with EGFR amplification respond favorably, and FISH-confirmed amplification can serve as a predictive marker. Nevertheless, *EGFR* amplification does not always correlate with protein overexpression, as detected using IHC, which complicates treatment decisions. Beyond its diagnostic relevance, the *EGFR amp/CDKN2A intact* profile was also associated with a poor prognosis. It independently predicted short-term DFS and OS in the multivariate analysis, underscoring its dual utility in diagnosis and risk stratification. A comparable molecular profile in non-small cell lung cancer has been linked to PD-L2-mediated immune evasion and an enhanced response to *EGFR*-tyrosine kinase inhibitor therapy combined with immune checkpoint inhibitors, suggesting that *EGFR/CDKN2A* co-alterations in OSCC may similarly modulate tumor immunogenicity and warrant further investigation [19].

In contrast, *EGFR*-negative OSCCs, which lack amplification and thus fall outside this subclassification, have no established molecular drivers or targeted therapies [40,41]. In our cohort, HRAS overexpression was observed in 60.6% of the *EGFR*-negative OSCC cases and was associated with reduced survival (Table 4). H-ras mutations have also been reported in oral carcinomas in other populations [42]. Despite the inconsistencies in prior studies, our findings support the role of HRAS in subclassifying this molecularly undefined subgroup [43]. HRAS likely functions as a key oncogenic driver in *EGFR*-negative OSCC and shapes distinct molecular and clinical phenotypes. As IHC cannot detect activating mutations, molecular sequencing is required to characterize HRAS-driven tumors. HRAS-positive OSCCs may represent an aggressive, *EGFR*-independent subtype amenable to targeted therapies, such as tipifarnib. HRAS activates the MAPK/ERK pathway and promotes invasion, proliferation, and resistance to apoptosis. Its activation may result from point mutations, overexpression, or posttranslational changes. Mutations such as Gly12Ala or Gly12Val stabilize the protein and are linked to strong IHC signals [22]. Several HRAS inhibitors are under development and may benefit patients with HRAS-positive and *EGFR*-negative OSCCs.

Our FISH analysis revealed additional alterations in *EGFR* and *CDKN2A* expression levels. OSCCs frequently showed *PIK3CA* amplification (29.4%) and deletions in *CCND1*, *TP53*, *PTEN*, and 1p36 locus (Figure 2), reflecting genomic instability. These co-alterations suggest that multiple driver events promote OSCC progression [44]. *EGFR^−^/HRAS^−^* OSCCs may constitute a distinct molecular subset. Multi-omics approaches, including epigenomic and transcriptomic profiling, are required to identify alternative drivers and refine the classification [45]. Notably, 10 OSCCs lacked alterations in the seven FISH-targeted genes, implicating other factors, such as HRAS, NOTCH1, or epigenetic mechanisms. The Cancer Genome Atlas and other studies have identified HRAS, CASP8, and NOTCH1 mutations in human papillomavirus-negative, *EGFR*-negative HNSCC [46]. Combining FISH with sequencing, copy number, and methylation analyses may improve OSCC subclassification and inform therapeutic strategies [47].

This study supports the feasibility of combining FISH and IHC for molecular stratification of OSCC. *EGFR* and CDKN2A *FISH* provides diagnostic and prognostic value, particularly the *EGFR amp/CDKN2A intact* profile, as a dual-purpose marker. HRAS IHC offers additional prognostic insights into *EGFR*-negative tumors. Both methods are applicable to FFPE tissues and are practical for routine pathology, including mid-sized labs [48,49]. Compared with next-generation sequencing, FISH is highly cost-effective for detecting gene copy alterations [50], whereas IHC reflects protein function and supports personalized care [51]. Similar to HER2 or ALK/ROS1 testing in other cancers, this approach holds clinical promise for OSCC [49,52]. The integration of these markers into diagnostic workflows may improve the molecular classification and risk assessment.

This study has some limitations. The single-institution nature of this study may have introduced a selection bias, particularly for a high proportion of patients with well-differentiated OSCC. The FISH method in FFPE tissue cannot reliably distinguish hemizygous from biallelic deletion because of nuclear truncation and probe resolution. Thus, our deletion definition may underestimate the complexity of allelic loss. Because EGFR amplification and CDKN2A deletion were mutually exclusive in our OSCC cohort, the independent effect of CDKN2A loss could not be assessed. Thus, the prognostic value of the *EGFR amp/CDKN2A intact* profile likely reflects EGFR amplification alone. Validation in larger cohorts, including tumors with both alterations, is needed to confirm this finding. HRAS IHC was limited to *EGFR*-negative cases to assess alternative pathways, which may have excluded overlapping alterations. HRAS IHC positivity does not necessarily indicate activating mutations. Future studies should include HRAS sequencing to validate IHC-based classifications and clarify mutation-specific significance. Alternative markers are needed to assess *EGFR*/HRAS double-negative OSCC. Prospective multicenter cohort and functional studies are warranted to confirm and extend these findings.

## 5. Conclusions

*EGFR* amplification was frequent in OSCC, and *CDKN2A* deletion was prevalent in OED, supporting their use as molecular markers for differential diagnoses. FISH for *EGFR/CDKN2A* and HRAS IHC can be used to stratify OSCC by diagnosis and prognosis, enabling practical molecular subclassification.

## Figures and Tables

**Table 1:** Clinicopathological characteristics of normal oral mucosa, OED, and OSCC.

Parameters		Normal Mucosa (n = 10) (%)	OED (n = 66) (%)	OSCC (n = 51) (%)
Sex	Male	5 (50.0)	21 (31.8)	29 (56.9)
	Female	5 (50.0)	45 (68.2)	22 (43.1)
Age (y)	Average (range)	57.0 (36–87)	69.9 (25–97)	58.9 (26–88)
Anatomical site	Tongue	2 (20.0)	22 (33.3)	30 (58.8)
	Gingiva	3 (30.0)	23 (34.8)	12 (23.5)
	Buccal mucosa	2 (20.0)	11 (16.7)	4 (7.9)
	Mouth floor	0	1 (1.5)	5 (9.8)
	Palate	3 (30.0)	5 (7.6)	0 (0)
	Lip	0	1 (1.5)	0 (0)
	Others	0	3 (4.6)	0 (0)
Epithelial dysplasia	Mild	-	21 (31.8)	-
	Moderate	-	14 (21.2)	-
	Severe/CIS	-	31 (47.0)	-
Histopathological grade	Well differentiated	-	-	42 (82.3)
	Moderately differentiated	-	-	8 (15.7)
	Poorly differentiated	-	-	1 (1.9)
Pathological tumor stage	T1	-	-	20 (39.2)
	T2	-	-	23 (45.1)
	T3	-	-	3 (5.9)
	T4a	-	-	5 (9.8)
	T4b	-	-	0 (0)
Pathological nodal stage	N0	-	-	33 (64.7)
	N1	-	-	9 (17.6)
	N2a	-	-	0 (0)
	N2b	-	-	8 (15.7)
	N2c	-	-	1 (2.0)
	N3	-	-	0 (0)
Neck dissection	Not performed	-	-	29 (56.9)
	performed	-	-	22 (43.1)
Clinical TNM stage	I	-	-	17 (33.3)
	II	-	-	12 (23.5)
	III	-	-	9 (17.7)
	IV	-	-	13 (25.5)
Ki-67 index	Average	-	12.48 (1–37.8)	18.23 (5.2–38.7)
*CCND1* amplification	Negative	10 (100.0)	53 (80.3)	38 (74.5)
	Positive	0 (0)	13 (19.7)	13 (25.5)
*CDKN2A* deletion	Negative	8 (80.0)	42 (63.6)	44(86.3)
	Positive	2 (20.0)	24 (36.4)	7(13.7)
*EGFR* amplification	Negative	10 (100.0)	60 (91.0)	36 (70.6)
	Positive	0 (0)	6 (9.0)	15 (29.4)
*PIK3CA* amplification	Negative	9 (90.0)	52 (78.8)	38 (74.5)
	Positive	1 (10.0)	14 (21.2)	13 (25.5)
*PTEN* deletion	Negative	10 (100.0)	54 (81.8)	42 (82.4)
	Positive	0 (0)	12 (18.2)	9 (17.6)
*TP53* deletion	Negative	10 (100.0)	39 (59.1)	37 (72.5)
	Positive	0 (0)	27 (40.9)	14 (27.5)
1p36 deletion	Negative	8(80.0)	37 (56.1)	40 (78.4)
	Positive	2 (20.0)	29 (43.9)	11 (21.6)

Note: - indicates “Not applicable.” OED, oral epithelial dysplasia; OSCC, oral squamous cell carcinoma; CIS, carcinoma in situ.

**Table 2:** Univariate and multivariate logistic regression analysis of diagnostic markers distinguishing OED from OSCC in 117 cases.

Parameters	OED (n = 66) (%)	OSCC (n = 51) (%)	Univariate OR (95% CI)	Univariate *p*	Multivariate OR (95% CI)	Multivariate *p*
*CDKN2A* gene deletion	24 (36.4)	7 (13.7)	0.28 (0.11–0.71)	0.006	-	-
*EGFR* gene amplification	6 (9.0)	15 (29.4)	4.17 (1.48–11.71)	0.007	-	-
*EGFR* amplification-positive/*CDKN2A* deletion-negative	2 (3.0)	15 (29.4)	13.33 (2.88–61.63)	<0.0001	10.9 (2.73–74.21)	0.0003
1p36 deletion	29 (43.9)	11 (21.6)	0.35 (0.15–0.80)	0.018	2.24 (0.90–5.86)	0.081
Ki-67 labeling index (%, mean)	12.48	18.23	1.10 (1.04–1.16)	0.001	1.08 (1.02–1.15)	0.004

Multivariate logistic regression was conducted including EGFR amplification-positive/CDKN2A deletion-negative, Ki-67 labeling index, and 1p36 locus deletion as covariates. -, Not included in the multivariate model. CI, confidence interval; OR, odds ratio; OED, oral epithelial dysplasia; OSCC, oral squamous cell carcinoma.

**Table 3:** Cox proportional hazards analyses for DFS and OS in 51 patients with OSCC.

	DFS				OS			
	Univariate		Multivariate		Univariate		Multivariate	
	HR (95% CI)	*p*	HR (95% CI)	*p*	HR (95% CI)	*p*	HR (95% CI)	*p*
Age (y)								
>60	2.13 (0.77–5.89)	0.170			0.99 (0.28–3.64)	0.980		
≤60								
Sex								
Male	0.72 (0.29–1.78)	0.480			1.13 (0.39–3.30)	0.820		
Female								
Tumor site								
Tongue	1.20 (0.47–3.06)	0.700			0.53 (0.11–2.64)	0.440		
Others								
Tumor size (cm)								
T1–T2	1.61 (1.38–17.51)	0.041	0.45 (0.12–1.67)	0.23	2.23 (1.81–6.74)	0.017	0.49 (0.07–3.24)	0.190
T3–T4								
Nodal status								
Positive	4.10 (1.37–12.27)	0.026	3.96 (1.05–15.02)	0.004	3.81 (1.14–12.82)	0.083		
Negative								
Histologic grade	1.69 (0.39–7.37)	0.320						
Well diff.					12.81 (1.07–15.38)	0.049	1.16 (0.27–4.94)	0.280
Moderate + poorly diff.								
*EGFR* amplification-positive/*CDKN2A* deletion-negative								
Positive	3.16 (1.28–7.83)	0.014	5.08 (1.36–18.96)	0.016 *	3.20 (1.10–9.33)	0.033	6.10 (1.36–10.69)	0.047 ^†^
Negative								
Clinical stage								
I, II	4.11 (1.47–11.48)	0.007	9.37 (2.11–41.66)	0.006 *	3.45 (1.94–12.71)	0.043	2.93 (0.78–11.04)	0.11 ^†^
III, IV								

* Both variables were retained as independent factors (*p* = 0.006 and 0.010, respectively). ^†^ Neither was retained (*p* = 0.11 and 0.069, respectively). DFS, disease-free survival; OS, overall survival; HR, hazard ratio; CI, confidence interval; diff., differentiated.

**Table 4:** Cox proportional hazards analyses for DFS and OS in 36 patients with EGFR amplification-negative OSCC.

	DFS				OS			
	Univari ate		Multivariate		Univariate		Multivariate	
	HR (95% CI)	*p*	HR (95% CI)	*p*	HR (95% CI)	*p*	HR (95% CI)	*p*
Age (y)								
>60	2.22 (0.59–8.29)	0.230			1.21 (0.30–7.4)	0.620		
≤60								
Sex								
Male	0.22 (0.01–0.89)	0.036	0.41 (0.08–2.02)	0.066	0.40 (0.073–2.22)	0.280		
Female								
Tumor site								
Tongue	2.18 (0.59–8.14)	0.250			0.53 (0.11–2.64)	0.440		
Others								
Tumor size (cm)								
T1–T2	0.34 (0.072–1.67)	0.150			5.83 (1.06–32.0)	0.035	3.12 (0.53–18.28)	0.190
T3–T4								
Nodal status								
Positive	17.4 (2.16–23.9)	<0.001	9.86 (1.22–79.60)	0.006	8.64 (1.01–74.07)	0.019	3.45 (0.38–31.35)	0.220
Negative								
Histologic grade	0.40 (0.097–1.57)	0.210			0.52 (0.095–2.85)	0.470		
Well diff.								
Moderate + poorly diff.								
HRAS IHC								
High	3.22 (1.85–8.91)	0.001	5.91 (1.09–19.63)	0.022 *	10.18 (1.78–12.91)	0.017	6.15 (1.36–10.69)	0.043 ^†^
Low								
Clinical stage								
I, II	3.05 (1.63–88.69)	0.002	6.11 (1.66–49.15)	0.035 *	6.83 (2.79–58.53)	0.038	3.18 (0.37–27.37)	0.29 ^†^
III, IV								

* Both clinical stage and HRAS IHC were retained as independent factors in the multivariate analysis (*p* = 0.035 and 0.028, respectively). ^†^ Neither clinical stage nor HRAS IHC was retained as an independent factor in the multivariate analysis (*p* = 0.29 and 0.06, respectively). DFS, disease-free survival; OS, overall survival; HR, hazard ratio; CI, confidence interval; IHC, immunohistochemistry; diff., differentiated.

## Data Availability

The original contributions presented in this study are included in the article/Supplementary Material. Further inquiries can be directed to the corresponding author.

## Supplementary Materials

The following supporting information can be downloaded at: https://www.mdpi.com/article/10.3390/cancers17243949/s1, Table S1: Correlation between genetic alterations and clinicopathological parameters in OED and OSCC; Table S2: Clinicopathological features stratified by HRAS expression in 36 EGFR-negative OSCC cases.

## Abbreviations

The following abbreviations are used in this manuscript:

AUC	Area under the curve
DFS	Disease-free survival
EGFR	Epidermal Growth Factor Receptor
FFPE	Formalin-fixed paraffin-embedded
FISH	Fluorescence in situ hybridization
HE	Hematoxylin–eosin
HNSCC	Head and neck squamous cell carcinoma
HR	Hazard ratio
OED	Oral epithelial dysplasia
OS	Overall survival
OSCC	Oral squamous cell carcinoma
ROC	Receiver operating characteristic

## References

[1] Tan Y., Wang Z., Xu M., Li B., Huang Z., Qin S., Nice E.C., Tang J., Huang C. (2023). Oral squamous cell carcinomas: State of the field and emerging directions. Int. J. Oral Sci..

[2] Johnson D.E., Burtness B., Leemans C.R., Lui V.W.Y., Bauman J.E., Grandis J.R. (2020). Head and neck squamous cell carcinoma. Nat. Rev. Dis. Primers.

[3] Wu C.S., Li H.P., Hsieh C.H., Lin Y.T., Yi-Feng Chang I.Y.K., Chung A.K., Huang Y., Ueng S.H., Hsiao Y.C., Chien K.Y. (2025). Integrated multi-omics analyses of oral squamous cell carcinoma reveal precision patient stratification and personalized treatment strategies. Cancer Lett..

[4] Davaatsend O., Altannamar M., Batbayar B., Jagdagsuren U. (2023). Factors influencing the 5-year survival rate of oral cancer patients in the Mongolian population: A retrospective cohort study. Front. Oral Health.

[5] Al-Hakami H.A., Al-Talhi A.A., AlRajhi B., Alshareef M.A., Awad B.I., Hussain T., Al-Garni M. (2025). Oncological outcomes, survival analysis, and failure patterns in patients with resectable squamous cell carcinoma of the oral tongue treated with glossectomy. Egypt. J. Otolaryngol..

[6] Aguirre-Urizar J.M., Lafuente-Ibáñez de Mendoza I., Warnakulasuriya S. (2021). Malignant transformation of oral leukoplakia: Systematic review and meta-analysis of the last 5 years. Oral Dis..

[7] Warnakulasuriya S., Ariyawardana A. (2016). Malignant transformation of oral leukoplakia: A systematic review of observational studies. J. Oral Pathol. Med..

[8] Ellonen R., Suominen A., Kelppe J., Willberg J., Rautava J., Laine H. (2023). Histopathological findings of oral epithelial dysplasias and their relation to malignant transformation. Cancer Treat. Res. Commun..

[9] Darling M.R., Hwang J.T.K., Dickson B.J., Cutz J.C., Salama S., McCord C., Pritzker K.P.H., Mock D., Thompson L.D.R. (2023). Assessing oral epithelial dysplasia risk for transformation to cancer: Comparison between histologic grading systems versus S100A7 immunohistochemical signature-based grading. Appl. Immunohistochem. Mol. Morphol..

[10] Monteiro L., Rocha E., Ferreira S., Salazar F., Pacheco J.J., Warnakulasuriya S. (2025). Tissue biomarkers for predicting the risk of oral cancer in patients diagnosed with oral leukoplakia: A systematic review of the past 4 years. J. Oral Pathol. Med..

[11] Ranganath K., Feng A.L., Franco R.A., Varvares M.A., Faquin W.C., Naunheim M.R., Saladi S.V. (2022). Molecular biomarkers of malignant transformation in head and neck dysplasia. Cancers.

[12] Jiang X., Ye J., Dong Z., Hu S., Xiao M. (2019). Novel genetic alterations and their impact on target therapy response in head and neck squamous cell carcinoma. Cancer Manag. Res..

[13] Chang J.Y.F., Tseng C.H., Lu P.H., Wang Y.P. (2021). Contemporary molecular analyses of malignant tumors for precision treatment and the implication in oral squamous cell carcinoma. J. Pers. Med..

[14] Li Q., Tie Y., Alu A., Ma X., Shi H. (2023). Targeted therapy for head and neck cancer: Signaling pathways and clinical studies. Signal Transduct. Target. Ther..

[15] Billard-Sandu C., Tao Y.G., Sablin M.P., Dumitrescu G., Billard D., Deutsch E. (2020). CDK4/6 inhibitors in P16/HPV16-negative squamous cell carcinoma of the head and neck. Eur. Arch. Otorhinolaryngol..

[16] Kang J.J., Ko A., Kil S.H., Mallen-St Clair J., Shin D.S., Wang M.B., Srivatsan E.S. (2023). EGFR pathway targeting drugs in head and neck cancer in the era of immunotherapy. Biochim. Biophys. Acta Rev. Cancer.

[17] Cabral L.G.S., Martins I.M., Paulo E.P.A., Pomini K.T., Poyet J.L., Maria D.A. (2025). Molecular mechanisms in the carcinogenesis of oral squamous cell carcinoma: A literature review. Biomolecules.

[18] Barem Rabenhorst S.H., Lima Verde Osterne R., Weege Nonaka C.F., Montezuma Sales Rodrigues A., Luiz Maia Nogueira R., Mário Rodriguez Burbano R., Barroso Cavalcante R. (2021). Detection of deletions in 1q25, 1p36 and 1pTEL and chromosome 17 aneuploidy in oral epithelial dysplasia and oral squamous cell carcinoma by fluorescence in situ hybridization (FISH). Oral Oncol..

[19] Wang S., Lai J.C., Li Y., Tang C., Lu J., Han M., Ye X., Jia L., Cui W., Yang J. (2025). Loss of CDKN2A enhances the efficacy of immunotherapy in EGFR-mutant non-small cell lung cancer. Cancer Res..

[20] Prior I.A., Hood F.E., Hartley J.L. (2020). The frequency of Ras mutations in cancer. Cancer Res..

[21] Ichimura N., Urata Y., Kobayashi T., Ebata R., Matsumoto H., Hibi H. (2024). Mutational landscape of Japanese patients with oral squamous cell carcinoma from comprehensive genomic profiling tests. Oral Oncol..

[22] Hamidavi Asl A., Shirkhoda M., Saffar H., Allameh A. (2023). Analysis of H-ras mutations and immunohistochemistry in recurrence cases of high-grade oral squamous cell carcinoma. Head Neck Pathol..

[23] WHO Classification of Tumours Editorial Board (2022). Head and Neck Tumours. WHO Classification of Tumours.

[24] Fukumura M., Ishibashi K., Nakaguro M., Nagao T., Saida K., Urano M., Tanigawa M., Hirai H., Yagyuu T., Kikuchi K. (2022). Salivary gland polymorphous adenocarcinoma: Clinicopathological features and gene alterations in 36 Japanese patients. J. Oral Pathol. Med..

[25] Mahmood H., Bradburn M., Rajpoot N., Islam N.M., Kujan O., Khurram S.A. (2022). Prediction of malignant transformation and recurrence of oral epithelial dysplasia using architectural and cytological feature specific prognostic models. Mod. Pathol..

[26] Warnakulasuriya S. (2001). Histological grading of oral epithelial dysplasia: Revisited. J. Pathol..

[27] Jäwert F., Fehr A., Öhman J., Stenman G., Kjeller G. (2022). Recurrent copy number alterations involving EGFR, CDKN2A, and CCND1 in oral premalignant lesions. J. Oral Pathol. Med..

[28] Sheu J.J.C., Hua C.H., Wan L., Lin Y.J., Lai M.T., Tseng H.C., Jinawath N., Tsai M.H., Chang N.W., Lin C.F. (2009). Functional genomic analysis identified epidermal growth factor receptor activation as the most common genetic event in oral squamous cell carcinoma. Cancer Res..

[29] Shahnavaz S.A., Bradley G., Regezi J.A., Thakker N., Gao L., Hogg D., Jordan R.C. (2001). Patterns of CDKN2A gene loss in sequential oral epithelial dysplasias and carcinomas. Cancer Res..

[30] Fiala C., Diamandis E.P. (2020). Mutations in normal tissues—Some diagnostic and clinical implications. BMC Med..

[31] Kennedy S.R., Zhang Y., Risques R.A. (2019). Cancer-associated mutations but no cancer: Insights into the early steps of carcinogenesis and implications for early cancer detection. Trends Cancer.

[32] Monteiro L., Delgado L., Amaral B., Ricardo S., Fraga M., Lopes C., Warnakulasuriya S. (2022). Occludin and claudin-1 are potential prognostic biomarkers in patients with oral squamous cell carcinomas: An observational study. Oral Surg. Oral Med. Oral Pathol. Oral Radiol..

[33] Loyo M., Li R.J., Bettegowda C., Pickering C.R., Frederick M.J., Myers J.N., Agrawal N. (2013). Lessons learned from next-generation sequencing in head and neck cancer. Head Neck.

[34] Kojima S., Kuribayashi N., Goda H., Nakashiro K.I., Uchida D. (2025). Oral cancer driver gene mutations in oral potentially malignant disorders: Clinical significance and diagnostic implications. Discov. Oncol..

[35] Califano J., van der Riet P., Westra W., Nawroz H., Clayman G., Piantadosi S., Corio R., Lee D., Greenberg B., Koch W. (1996). Genetic progression model for head and neck cancer: Implications for field cancerization. Cancer Res..

[36] Rane J.K., Frankell A.M., Weeden C.E., Swanton C. (2023). Clonal evolution in healthy and premalignant tissues: Implications for early cancer interception strategies. Cancer Prev. Res..

[37] John E., Lesluyes T., Baker T.M., Tarabichi M., Gillenwater A., Wang J.R., Van Loo P., Zhao X. (2024). Reconstructing oral cavity tumor evolution through brush biopsy. Sci. Rep..

[38] Adorno-Farias D., Santos J.N.D., González-Arriagada W., Tarquinio S., Santibáñez Palominos R.A., Martín Martín A.J.M., Fernandez-Ramires R. (2023). Whole-exome sequencing of oral epithelial dysplasia samples reveals an association with new genes. Braz. Oral Res..

[39] Chung C.H., Li J., Steuer C.E., Bhateja P., Johnson M., Masannat J., Poole M.I., Song F., Hernandez-Prera J.C., Molina H. (2022). Phase II multi-institutional clinical trial result of concurrent cetuximab and nivolumab in recurrent and/or metastatic head and neck squamous cell carcinoma. Clin. Cancer Res..

[40] Leemans C.R., Snijders P.J.F., Brakenhoff R.H. (2018). The molecular landscape of head and neck cancer. Nat. Rev. Cancer.

[41] Huang S.F., Cheng S.D., Chien H.T., Liao C.T., Chen I.H., Wang H.M., Chuang W.Y., Wang C.Y., Hsieh L.L. (2012). Relationship between epidermal growth factor receptor gene copy number and protein expression in oral cavity squamous cell carcinoma. Oral Oncol..

[42] Saranath D., Chang S.E., Bhoite L.T., Panchal R.G., Kerr I.B., Mehta A.R., Johnson N.W., Deo M.G. (1991). High frequency mutation in codons 12 and 61 of H-ras oncogene in chewing tobacco-related human oral carcinoma in India. Br. J. Cancer.

[43] Devi P., Dwivedi R., Sankar R., Jain A., Gupta S., Gupta S. (2024). Unraveling the genetic web: H-Ras expression and mutation in oral squamous cell carcinoma—A systematic review. Head Neck Pathol..

[44] Grossmann P., Cristea S., Beerenwinkel N. (2020). Clonal evolution driven by superdriver mutations. BMC Evol. Biol..

[45] Liu X., Liu Y., Tian X., Xi Y., Lu M., Zou X., Chen W. (2025). A novel molecular classification system for head and neck squamous cell carcinoma: Predicting treatment response and metastatic potential through multi-omics analysis. Discov. Oncol..

[46] Cancer Genome Atlas Network (2015). Comprehensive genomic characterization of head and neck squamous cell carcinomas. Nature.

[47] Bhosale P.G., Cristea S., Ambatipudi S., Desai R.S., Kumar R., Patil A., Kane S., Borges A.M., Schäffer A.A., Beerenwinkel N. (2017). Chromosomal alterations and gene expression changes associated with the progression of leukoplakia to advanced gingivobuccal cancer. Transl. Oncol..

[48] Huber D., Kaigala G.V. (2018). Rapid micro fluorescence in situ hybridization in tissue sections. Biomicrofluidics.

[49] Wolff A.C., Hammond M.E.H., Allison K.H., Harvey B.E., Mangu P.B., Bartlett J.M.S., Bilous M., Ellis I.O., Fitzgibbons P., Hanna W. (2018). Human epidermal growth factor receptor 2 testing in breast cancer: American Society of Clinical Oncology/College of American Pathologists Clinical Practice Guideline Focused Update. J. Clin. Oncol..

[50] Nelhűbel G.A., Cserepes M., Szabó B., Türk D., Kárpáti A., Kenessey I., Rásó E., Barbai T., Hegedűs Z., László V. (2021). EGFR alterations influence the cetuximab treatment response and c-MET tyrosine-kinase inhibitor sensitivity in experimental head and neck squamous cell carcinomas. Pathol. Oncol. Res..

[51] Sheffield B.S. (2016). Immunohistochemistry as a practical tool in molecular pathology. Arch. Pathol. Lab. Med..

[52] Mayer C., Ofek E., Fridrich D.E., Molchanov Y., Yacobi R., Gazy I., Hayun I., Zalach J., Paz-Yaacov N., Barshack I. (2022). Direct identification of ALK and ROS1 fusions in non-small cell lung cancer from hematoxylin and eosin-stained slides using deep learning algorithms. Mod. Pathol..

